# Investigating Cardiac Morphological Alterations in a Pentylenetetrazol-Kindling Model of Epilepsy

**DOI:** 10.3390/diagnostics10060388

**Published:** 2020-06-09

**Authors:** Enes Akyuz, Kristina Polat, Sukru Ates, Demet Unalmis, Adem Tokpinar, Seher Yilmaz, Emin Kaymak, Zuleyha Doganyigit, Chiara Villa

**Affiliations:** 1 Department of Biophysics, Faculty of Medicine, Yozgat Bozok University, 66100 Yozgat, Turkey; 2 Faculty of Medicine, Yozgat Bozok University, 66100 Yozgat, Turkey; Polatkristina@gmail.com; 3 Department of Anatomy, Faculty of Medicine, Yozgat Bozok University, 66100 Yozgat, Turkey; sukru.ates@yobu.edu.tr (S.A.); demetunalmis@gmail.com (D.U.); adem.tokpinar@yobu.edu.tr (A.T.); seher.yilmaz@yobu.edu.tr (S.Y.); 4 Department of Histology and Embriology, Faculty of Medicine, Yozgat Bozok University, 66100 Yozgat, Turkey; e_kaymak@hotmail.com (E.K.); zuleyha.doganyigit@gmail.com (Z.D.); 5 School of Medicine and Surgery, University of Milano-Bicocca, 20900 Monza, Italy

**Keywords:** SUDEP, epilepsy, pentylenetetrazol, heart, electrocardiographic recordings

## Abstract

Epilepsy is a group of neurological disorders characterized by abnormal electrical activity in the central nervous system (CNS) and recurrent seizures representing the principal clinical manifestation. Sudden unexpected death in epilepsy (SUDEP) is the predominant cause of death in young epileptic patients. SUDEP patients displayed an increased cardiovascular (CV) risk, probably due to an impaired autonomic control of CV functions, but the underlying mechanisms need to be explored yet. Therefore, we aimed to examine the cardiac morphological alterations in a pentylenetetrazol (PTZ)-kindled rat model, a well-established tool for studying chronic epilepsy. To complete this, the distance between the atria, between the atrium and ventricle were measured, the heart was weighed, and the pathological morphology of dissected hearts was analyzed by histological assessment with hematoxylin and eosin staining. A significantly decreased distance between atria and a significant increase in heart weight were observed in PTZ-kindled rats which interestingly also displayed increased hemorrhagic content when compared with controls. Our findings provided evidence that changes in cardiac morphology may be related to autonomic CV dysfunctions occurring during SUDEP while also opening up more avenues to better develop novel drugs for the treatment of this disorder.

## 1. Introduction

Epilepsy is a group of chronic neurological disorders with a complex etiology and represents one of the most common serious brain conditions, affecting over 70 million people worldwide [1]. It is clinically characterized by recurrent seizures caused by an imbalance between cerebral excitability and inhibition in the central nervous system (CNS) which may either affect specific brain systems or originate in a restricted area and spread to involve multiple cortical and subcortical circuits [2]. Sudden unexpected death in epilepsy (SUDEP) is the most common cause of death in young adults with refractory epilepsy having an incidence rate of 24-fold higher than control populations and accounting for an average of 17% of deaths in patients with epilepsy [3]. Most cases occur during sleep with the patient in the prone position (which could aggravate seizure-related respiratory dysfunction) and are unwitnessed, though there is often evidence of a recent seizure at the time of death [4]. The majority of documented cases displayed generalized tonic-clonic seizures, which represent the most important risk factor for SUDEP. Therefore, an effective treatment with appropriate antiepileptic drugs (AEDs) has been well-documented in preventing SUDEP [5]. Increasing evidence reports that seizures can alter the autonomic nervous system control of cardiorespiratory function (neurocardio-respiratory connection), leading to hypoventilation, apnea, and cardiovascular (CV) collapse [6,7]. Despite the etiology of SUDEP remaining elusive, the underlying cardiac pathology accounts for the majority of sudden cardiac deaths [7,8]. Different arrhythmias have been described either occurring during (ictal) or after (postictal) seizures; ictal asystole, bradycardia, and AV block are the most frequent. Moreover, the presence of CV diseases seems to be a significant contributor to the increased mortality in epileptic people compared to the general population. Epidemiological studies have largely reported that people with epilepsy show a higher prevalence of structural cardiac disorder than those without epilepsy [9]. There is evidence for increased cardiac autonomic stimulation in patients with SUDEP by having more marked in epileptic seizures during sleep. Its recurrent stimulation may cause structural damage to the heart seen through myocardial fibrosis already being observed in patients with SUDEP [10]. In an animal model of epilepsy, cardiac hypertrophy appears to be related to autonomic changes that arise from seizures, suggesting that the heart could structurally be affected by seizures [11].

Here, we aimed to investigate possible changes of heart morphology accompanying SUDEP, using a kindling rat model of epilepsy. In this well-established tool, kindling is chemically induced by an intraperitoneally (i.p.) injection of pentylenetetrazol (PTZ), a GABA_A_ receptor antagonist known to cause a persistent increase in seizure liability with minimal neuronal damage [12].

## 2. Materials and Methods

### 2.1. Animals

Wistar albino rats (280–350 g) were obtained from the Research Center of Kayseri Erciyes University. They were housed in a controlled environment with a temperature of 24 ± 2 °C and humidity of 60% under a 12-h light/dark cycle. Animals were given free access to tap water and standard food ad libitum. All procedures were carried out in strict accordance with the recommendations in the Guide for the Care and Use of Laboratory Animals adopted by the National Institutes of Health (NIH, Bethesda, MD, USA) and the Declaration of Helsinki. The experimental protocol of this study was approved by the Animal Ethics Committee of the Kayseri Erciyes University (ethics committee decision number: 2019/027, approved 13 February 2019). All efforts were made to minimize animal suffering.

### 2.2. Chemicals and Experimental Design

**Figure 1:**
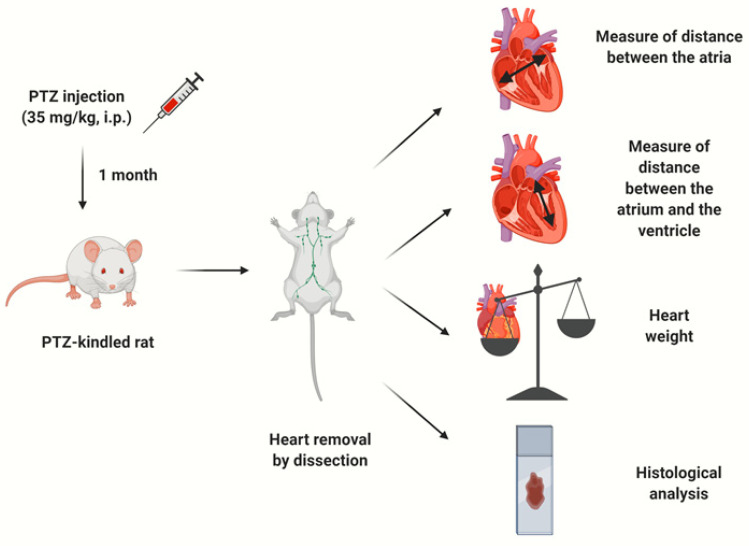
A summary of the experimental study: pentylenetetrazol (PTZ) injection, heart dissection, targeted measures and histological analysis, respectively.

PTZ (P6500, Sigma-Aldrich, St. Louis, MO, USA) was dissolved in isotonic saline (0.9% NaCl) and freshly prepared on the day of the administration. Rats were randomly divided into: (i) a control group that received 0.9% saline (*n* = 14; 7 males and 7 females) and (ii) a PTZ kindling epilepsy group injected with 35 mg/kg of PTZ (*n* = 14; 7 males and 7 females). An overview of the complete experimental design is summarized in Figure 1.

### 2.3. PTZ-Induced Kindling Model

The PTZ kindling epilepsy model was induced as previously described [13]. Briefly, rats were injected with a subconvulsive dose of PTZ (35 mg/kg, i.p.) three times a week (Monday, Wednesday, and Friday) for one month until the generation of kindling. After each injection, the rats were placed alone in an isolated transparent Plexiglas cage and their convulsive behavior was video recorded for a period of 30 min. The records were analyzed blindly for the seizure’s stage, the latency of the seizure onset, and the duration of the seizure. The severity of seizures was scored according to the well-established Racine’s scoring which is as follows: stage 0, no response; stage 1, facial movements, ear, and whisker twitching; stage 2, myoclonic convulsions without rearing; stage 3, myoclonic jerks, upright position with clonic forelimb convulsions; stage 4, clonic-tonic convulsions; stage 5, generalized clonic-tonic seizures with loss of postural control; stage 6, death. Full kindling was achieved when the rat showed stage 4 or 5 after three consecutive injections of PTZ [14].

### 2.4. Heart Dissection and Measurements

**Figure 2:**
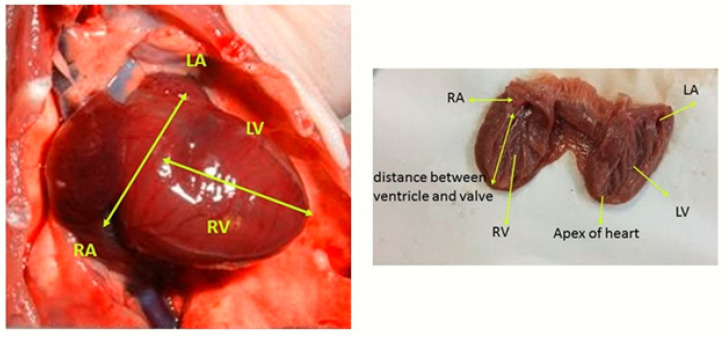
Representative pictures displaying the anatomical measured distances in the heart of rats (RA: Right Atrium, LA: left Atrium, RV: Right Ventricle, LV: Left Ventricle).

Once the animals became fully kindled, the rats were decapitated and the chest wall was cleaned. The left cartilage was cut at the level of the ribs and the chest cavity was opened. In order to identify morphological structures and record changes, the entire heart was viewed for its anatomical position and evaluated for dextrocardia. No change in anatomical position was observed in animals belonging to the experimental groups. In animals with chest cavity, the heart was measured by an electronic caliper with a measuring range of 0–150 mm. The distance between the two atria (right atrium and left atrium) was measured as the distance between the largest atria in the direction of a line that passes in the lower half of the septum and just above the foramen venae cavae inferioris. Then, atrioventricular distance was measured by electronic caliper in line with a line drawn from the lower edge of the valva seminularis aorta to the apex cordis. After the measures were completed, the heart was removed by cutting the aorta and the upper edge of the truncus pulmonalis. The hearts cleaned from the surrounding structures were weighed with a sensitive scale with a sensitivity of 0.001 gr and weight measurements were completed. All measurements were made by the same person with the same caliper and precision scales. The determined points of the measurements are shown in Figure 2.

### 2.5. Hematoxylin and Eosin (HE) Staining

Heart tissues were fixed with 4% paraformaldehyde for 48 h, embedded in paraffin wax, and then sectioned at 5–6 μm thickness using a cryostat. Sections were deparaffinized by immersion in xylene and stained with hematoxylin for 5 min. After being washed, sections were stained with eosin for 5 min and incubated in an ascending alcohol concentration gradient (70, 80, and 90%) for 5 min. Heart slices were then placed in 100% alcohol for 5 min and twice in xylene for 1 min. After being covered with Entellan, specimens were evaluated under an Olympus BX53 microscope (Olympus, Tokyo, and Japan) and the images were captured with a DP71 camera. Sections were taken from all cardiac tissue and seven slides from each group were examined. Scoring was performed by examining 35 nonoverlapping microscope fields obtained from five different sections of heart tissue. For quantification, the images were analyzed by color segmentation plugin–ImageJ software (NIH, Bethesda, MD, USA). The hemorrhagic content was calculated as percentage of hemorrhagic area/total examined area. The measurement of cardiomyocyte length was evaluated by analyzing 40 cells at different location for each slide.

### 2.6. Statistical Analysis

Data are given as mean values ± standard error of the mean (SEM). Shapiro–Wilk test was used to check a normal distribution of data in each group. Comparisons between the PTZ-kindled and control groups were analyzed by an unpaired two-tailed Student’s *t*-test. Version 18 of Statistical Package for Social Science (SPSS 18, IBM Corporation, Chicago, IL, USA) was used for statistical analysis of the data. Differences were considered significant at * *p* < 0.05.

## 3. Results

### 3.1. PTZ Induced Seizures in Rats

**Figure 3:**
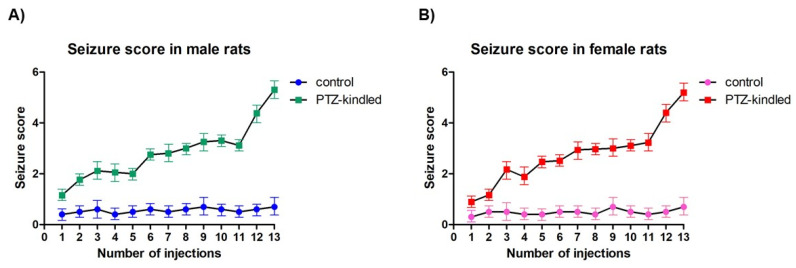
Racing score exhibited by male (**A**) and female (**B**) rats after each injection of PTZ or saline (control). Values are presented as the mean ± SEM, *n* = 7 for each group.

Rats in the control group displayed no abnormal behavior after an injection of saline; they had normal appetites and exhibited no seizures throughout the entire experimental procedure. Starting from the 2nd to 7th injections after the start of PTZ administration, rats began to show epileptic symptoms, including nodding, gazing, facial twitching, scratching, and forelimb−limited seizures. Between the 3rd and 4th injection, six deaths occurred in the PTZ-treated group (males: *n* = 3; females: *n* = 3) because of the constant status epilepticus, so they were excluded from the subsequent analysis and new animals were added instead of dead ones in order to maintain the total number of PTZ-kindled rats. At the 13th injection, widespread muscle spasms and generalized tonic-clonic seizures were displayed in both male and female rats (5.31 ± 0.58, Figure 3A; 5.2 ± 0.47, Figure 3B, respectively). Therefore, the model of chronic epilepsy induced by PTZ kindling was successfully established in the 14 rats of the PTZ-treated group. PTZ-kindled male rats had their first seizure in 308.78 ± 95.15 s while the female rats in 266.03 ± 66.12 s. Seizures usually occurred 2−10 min after PTZ injection with a duration of 2−5 min per episode. No significant changes in the body weights of rats were found during the PTZ injection process (data not shown).

### 3.2. PTZ-Kindled Rats Exhibited Lower Distance between Atria and Showed Heavier Heart Weight

**Figure 4:**
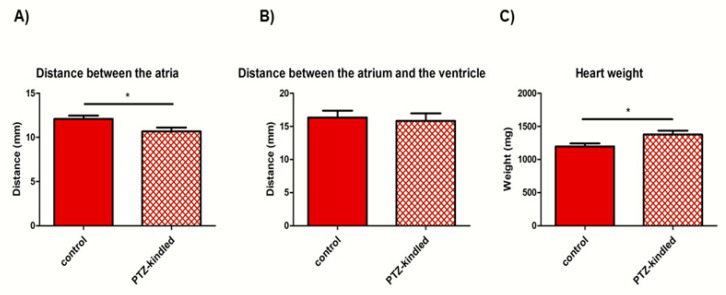
Measures of distance between the atria (**A**), between the atrium and the ventricle (**B**), and heart weight (**C**) in PTZ-kindled rats vs. controls. Data represent the mean ± SEM of the distance expressed as mm and the heart weight expressed as mg, *n* = 14 for each group, * *p* < 0.05.

PTZ-kindled rats showed a decreased distance between the atria as compared with controls (10.68 ± 0.44 mm vs. 12.09 ± 0.38 mm, * *p* < 0.05, Figure 4A) whereas no differences were found in the distance between the atrium and the ventricle (15.83 ± 1.12 mm vs. 16.34 ± 1.03 mm, *p* > 0.05, Figure 4B). Furthermore, an increase in heart weight was found in PTZ-kindled rats compared to the control group (1377.63 ± 59.49 mg vs. 1198.17 ± 44.00 mg, * *p* < 0.05, Figure 4C). All data are summarized into Table 1.

### 3.3. Histological Analysis Revealed Hemorrhagic Areas in Cardiac Tissue and an Increase of Cardiomyocyte Length in PTZ-Kindled Rats

**Figure 5:**
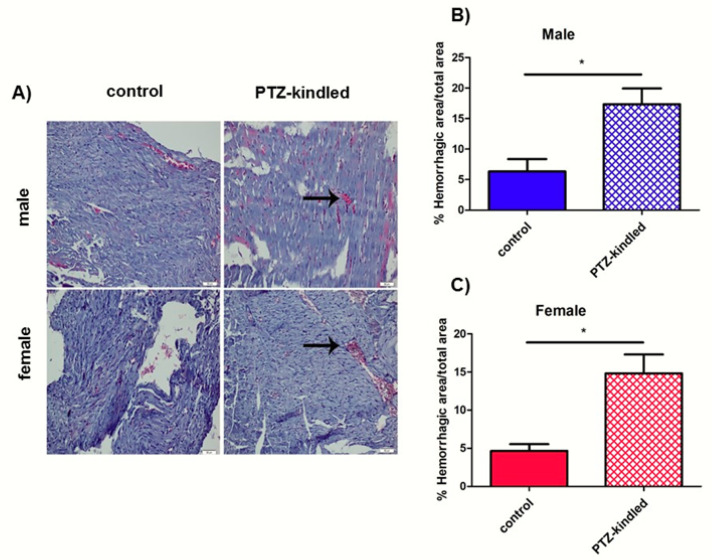
(**A**) Representative images of Hematoxylin and Eosin (HE)-stained heart sections from both male and female PTZ-kindled rat rats vs. controls. Hemorrhagic areas are indicated by arrows. Pictures were taken at the magnification of ×200. Scale bar: 50 μm. Bars represent the mean ± SEM of the percentage of the hemorrhagic area in male (**B**) and female (**C**) rats, *n* = 7 for each group, * *p* < 0.05.

With the purpose of investigating morphological changes in heart tissue, we performed histological analysis. HE staining showed a significant increase in hemorrhagic content in both male and female PTZ-kindled rats as compared with controls (2.74-fold change and 3.21-fold change over controls, respectively, * *p* < 0.05, Figure 5). Moreover, an increase in cardiomyocyte length was observed in PTZ-kindled rats as compared with controls, although the statistical significance was reached only in the female group (39.12 ± 3.71 μm vs. 37.22 ± 2.97 μm, *p* > 0.05; 43.46 ± 3.02 μm vs. 36.05 ± 3.64 μm, * *p* < 0.05, respectively, Table 2).

## 4. Discussion

SUDEP represents the major cause of epilepsy-related mortality, which targets primarily young people. Pathophysiological mechanisms of SUDEP are likely to be heterogeneous and may be multifactorial. It has been hypothesized that ictal activity that arises in or spreads to the autonomic nervous system can disrupt the functional connectivity of this network by activating or inhibiting the autonomic areas, leading to different autonomic manifestations, including CV dysfunctions [3]. In particular, cardiac rhythm and conduction abnormalities are common in epileptic patients, mainly if the seizure is prolonged or generalized [15].

An impairment of the autonomic nervous system in the regulation of CV functions during seizures has been recently reported by our previous study performed in a PTZ-kindled rat model, showing aberrant blood pressure and electrocardiographic (ECG) recordings [16]. We suggested that this data may be related to an altered expression of inwardly rectifying potassium (Kir) channels in the heart [17]. In this regard, Kir channels play a key role in cardiac excitability and they have been proposed to explain autonomic CV dysfunctions, observed also in multiple sclerosis [18]. Indeed, emerging evidenced shows that cardiac ion channels expression is altered in animal models of both genetic and acquired forms of epilepsy. Their dysregulation could represent a consequence of seizure activity, leading to an electrophysiological dysfunction of the heart [19]. In light of these considerations, we investigated possible structural cardiac alterations accompanying epilepsy. In the present study, we found a decreased distance between the atria and an increase in both heart weight and cardiomyocyte length in PTZ-kindled rats, indicative of a significant change in cardiac morphology. The distance between the atria affects the ventricular filling and may cause hypertrophy in the ventricular muscles, leading to various heart disorders. We can speculate that if distance between the atria is shortened, the time required for the atria to fill up is not expected, as depolarization will be initiated. Since enough blood does not accumulate in the heart, its pressure decreases. In addition, as the myocardium is remodeled in response to a continuous increase in stress and/or injury in an effort to normalize stress of myocardial wall, we can hypothesize that the increase of both heart weight and cardiomyocyte size should be related to muscle hypertrophy. According to these findings, Damasceno and collaborators found an increase in the duration of ischemia/reperfusion arrhythmia and cystic artery pressure, a prolongation of QRS wave, and hypertrophy in the heart of epileptic rats [20]. It has also been reported that the most common concomitant heart pathologies in SUDEP cases are myocyte hypertrophy and myocardial fibrosis [21]. In a similar study, histological examinations of the ventricular myocardium performed 48 h after kainic acid administration showed development of hypercontraction, band necrosis, inflammatory cell infiltration, and edema [22]. Moreover, a higher left ventricle stiffness, left ventricle filling pressures, and a greater left atrial volume have been found in epileptic patients without any known CV disease. The authors argued that stiffness is related to fibrosis through extracellular matrix deposition, which leads to systolic/diastolic dysfunction and arrhythmogenesis [23]. Our results also demonstrated the presence of hemorrhagic content in the cardiac tissue of PTZ-kindled rats. We can speculate that multiple and recurrent seizures cause accumulated damage in cardiac tissue which in turn has the potential to interfere with electrical conduction in the heart, leading thus to SUDEP during a typical tonic-clonic seizure. Hemorrhage results in a diminished return of venous blood to the heart, inducing a lowering of blood pressure as already demonstrated by our previous study conducted in the same in vivo model during an ictal seizure [16]. However, our results may be eventually attributed to a secondary toxic effect on myocardium related to PTZ exposure. Indeed, it has been shown that PTZ altered the density and sensitivity of different glutamate receptor subtypes in several brain regions [24], leading to an enhancement in susceptibility of the glutamatergic transmission systems that resulted in glutamate excitotoxicity. Thus, we cannot exclude that PTZ may also affect the glutamate receptors located on myocardium. Although there is no experimental model that reproduces faithfully all features of human epilepsy, multiple administrations of PTZ are largely used to develop a chronic epilepsy model mimicking recurrent generalized tonic-clonic seizures and, thus, to identifying key events underlying epileptogenic mechanisms. Alternatively to other time-consuming and costly epilepsy models, it has several advantages, including a precise record of the time seizure onset and the development of their severity/intensity in time. However, as compared to electrical kindling, PTZ-kindled model has less experimental control of the timings of seizure evocation and a delay between the drug delivery and when the seizure onset occurs [25]. As additional limitations of this model, adult animals may show mossy fiber sprouting and do not present with spontaneous seizures [26]. Nevertheless, it has been reported that repetitive PTZ-induced seizures led to an overexpression of P-gp protein that is associated with membrane depolarization in the hippocampus and with altered cardiac rhythm in the heart. Interestingly, both of them are related with SUDEP pathogenesis [27].

To the best of our knowledge, this is the first study investigating alterations in cardiac morphology in terms of a distance difference of cardiac structure in SUDEP. Although the pathogenesis is not completely clarified, our data may help to better understand the molecular mechanisms underneath CV complications in epilepsy and to develop novel drugs for the treatment of this disorder. Moreover, CV comorbidities will provide insight into shared pathways for epilepsy and give a window into common genetic predispositions.

## 5. Conclusions

In summary, our results provide evidence that changes in cardiac morphology may be related to autonomic CV dysfunctions in epilepsy. SUDEP remains the most common cause of epilepsy-related death among epileptic children and young adults, but the pathophysiology of SUDEP is still poorly understood and we lack any established preventive strategy other than seizure control. However, our results could be predictive of SUDEP risk according to the presence of the spontaneous death of animals during the treatment with PTZ. Thus, identifying the structural cardiac pathologies may provide more insight into the heterogeneous SUDEP mechanisms. In this regard, PTZ-kindled model will be useful for a rapid screening of AEDs which do not alter our assessed cardiac parameters, as a result of impaired autonomic system. Epileptic patients may be predisposed to cardiac arrhythmias due to the effects of the recurrent seizure on cardiac microstructure. Therefore, the ability to stratify the SUDEP risk on the basis of interictal autonomic parameters could have valuable prognostic implications.

## Figures and Tables

**Table 1:** A summary of results regarding measures of distance between the atria, between the atrium and the ventricle, and heart weight in PTZ-kindled rats vs. controls.

Measured Parameters	Control	PTZ-Kindled	*p* Value
Distance between the atria	12.09 ± 0.38 mm	10.68 ± 0.44 mm	0.032 *
Distance between the atrium and the ventricle	16.34 ± 1.03 mm	15.83 ± 1.12 mm	0.753
Heart weight	1198.17 ± 44.00 mg	1377.63 ± 59.49 mg	0.036 *

Data are expressed as the mean ± SEM, * *p* < 0.05.

**Table 2:** Measures of cardiomyocyte length PTZ-kindled rats vs. controls.

Groups	Control	PTZ-Kindled	*p* Value
Male	37.22 ± 2.97 μm	39.12 ± 3.71 μm	0.140
Female	36.05 ± 3.61 μm	43.46 ± 3.02 μm	0.004 *

Data are expressed as the mean ± SEM, * *p* < 0.05.
